# A nucleotide-dependent oligomerization of the *Escherichia coli* replication initiator DnaA requires residue His136 for remodeling of the chromosomal origin

**DOI:** 10.1093/nar/gkz939

**Published:** 2019-10-29

**Authors:** Rahul Saxena, Christopher B Stanley, Pankaj Kumar, Matthew J Cuneo, Digvijay Patil, Jyoti Jha, Kevin L Weiss, Dhruba K Chattoraj, Elliott Crooke

**Affiliations:** 1 Department of Biochemistry and Molecular & Cellular Biology, Georgetown University Medical Center, Washington, DC 20007, USA; 2 Computational Sciences and Engineering Division, Oak Ridge National Laboratory, Oak Ridge, TN 37831, USA; 3 Department of Biochemistry, Jamia Hamdard University, Delhi 110062, India; 4 Department of Structural Biology, St. Jude Children's Research Hospital, Memphis, Tennessee 38105, USA; 5 Basic Research Laboratory, Center for Cancer Research, National Cancer Institute, National Institutes of Health, Bethesda, Maryland, USA; 6 Neutron Scattering Division, Oak Ridge National Laboratory, Oak Ridge, TN 37831, USA; 7 Lombardi Comprehensive Cancer Center, Georgetown University Medical Center, Washington, DC 20007, USA

## Abstract

*Escherichia coli* replication initiator protein DnaA binds ATP with high affinity but the amount of ATP required to initiate replication greatly exceeds the amount required for binding. Previously, we showed that ATP-DnaA, not ADP-DnaA, undergoes a conformational change at the higher nucleotide concentration, which allows DnaA oligomerization at the replication origin but the association state remains unclear. Here, we used Small Angle X-ray Scattering (SAXS) to investigate oligomerization of DnaA in solution. Whereas ADP-DnaA was predominantly monomeric, AMP–PNP–DnaA (a non-hydrolysable ATP-analog bound-DnaA) was oligomeric, primarily dimeric. Functional studies using DnaA mutants revealed that DnaA(H136Q) is defective in initiating replication *in vivo*. The mutant retains high-affinity ATP binding, but was defective in producing replication-competent initiation complexes. Docking of ATP on a structure of *E. coli* DnaA, modeled upon the crystallographic structure of *Aquifex aeolicus* DnaA, predicts a hydrogen bond between ATP and imidazole ring of His136, which is disrupted when Gln is present at position 136. SAXS performed on AMP–PNP–DnaA (H136Q) indicates that the protein has lost its ability to form oligomers. These results show the importance of high ATP in DnaA oligomerization and its dependence on the His136 residue.

## INTRODUCTION

The recognition of the chromosomal origin of replication (*oriC*) in *Escherichia coli* requires the initiator protein DnaA (1,2). DnaA protein is a member of the AAA+ (ATPase associated with cellular functions) super-family of proteins that includes Cdc6 and some of the origin recognition complex (ORC) proteins involved in initiation of eukaryotic DNA replication (3–4). In *E. coli*, DnaA protein is tightly associated with ATP and ADP (*K*_D_ = 0.03 μM and 0.1 μM, respectively) (2). Whereas both ADP-DnaA and ATP-DnaA interact with specific regions within *oriC* (5), only ATP-DnaA stimulates the opening of the DNA duplex at AT-rich 13-mer DNA unwinding elements (6,7). Once DnaA has unwound the duplex, DnaB helicase and subsequently the rest of the replisome components can be loaded onto the origin, so that the replication can commence (8,9).

*Escherichia coli* DnaA contains four functional domains, I-IV (10,11). Domain I is important for DnaA oligomer formation (12,13), DnaB helicase loading (14–16), and interactions with other protein partners (17–19). Domain II is an unstructured flexible region that is thought to align domain I with domains III and IV (20). It may also play a role in stable binding of DnaB helicase with the initiator protein (21). Domain III is the most conserved region and contains the characteristic features of AAA+ protein family members, including Walker A, Walker B, Sensor I and Sensor II motifs (22,23). Lastly, domain IV is required for sequence-specific DnaA binding at several chromosomal loci (24–26), including *oriC* (27).

The accumulation of multiple DnaA molecules on *oriC* is a pre-requisite for successful initiation. However, the stoichiometry of DnaA molecules bound per origin is still debatable (1,2,28–30). Several studies have revealed that the structure of *oriC* is complex, containing both ‘high-affinity’ sites, such as R1, R2, & R4 as well as ‘low-affinity’ sites, such as I1 and I2 (5,31–33). Binding of ADP-DnaA and/or ATP-DnaA to high-affinity sites generates the initial bacterial origin recognition complex (bORC) (31–33). Further binding preferentially of ATP-DnaA to low-affinity sites (31–33) directs the assembly of higher-order DnaA oligomerization at *oriC*, producing replication-competent pre-replication complexes (pre-RC) (34).

That DnaA oligomerizes in solution is supported by glutaraldehyde crosslinking (35), where DnaA bound to ATP or non-hydrolyzable ATP-analogues formed higher order complexes when compared to DnaA bound to ADP. Although the X-ray crystal structure of *E. coli* DnaA protein still has not been reported, crystal structure of truncated *Aquifex aeolicus* DnaA (domains III- IV) bound to ADP or AMPPCP are available (22,23). Whereas the truncated DnaA protein crystals bound to ADP are uniformly composed of monomers (22), the crystals of AMPPCP-DnaA contain both monomer and tetramer structures (23). Taken together these data indicate a distinct role of ATP as opposed to ADP on the oligomerization of DnaA. However, whether stable DnaA oligomers form in solution that act as building blocks for the nucleoprotein assembly at *oriC* remains an open question. Another major unknown is the requirement of high concentration of ATP for origin opening, about several order of magnitude higher than that suffices for DnaA binding to low-affinity sites (33).

To address these questions, here we have used the Small Angle X-ray Scattering (SAXS), which can delineate the oligomerization state of a protein in solution (36,37). Our results show that high ATP concentrations are required for DnaA oligomerization, primarily to the dimeric form. Site-directed mutagenesis, followed by functional and molecular modeling studies, revealed that the His136 residue, present within the AAA+ domain of DnaA, plays an important role in DnaA dimerization and making the origin competent for replication.

## MATERIALS AND METHODS

### Enzymes, chemicals and oligonucleotides

Enzymes for DNA cloning were purchased from New England Biolabs. Ingredients for buffers and LB medium used were purchased from Vita Scientific. Reagents and kits used for the preparation of plasmid DNA, purification of PCR amplified DNA fragments and purification of radiolabeled primers were purchased from Qiagen. Nickel–nitrilotriacetic acid–agarose matrix (Ni-NTA agarose) for purification of recombinant histidine-tagged proteins was purchased from Qiagen. Reagents for preparing sequencing gels to analyze protein–DNA interactions were purchased from National Diagnostics. Radioactive isotopes [γ-^32^P]ATP (3000 Ci/mmol) and [α-^32^P]ATP (3000 Ci/mmol) were purchased from Perkin-Elmer Life Sciences. DNA oligonucleotides (Supplementary Table S1) were designed using gene runner software and custom synthesized by Integrated DNA Technologies. Mutations incorporated within *dnaA* were confirmed by Sanger sequencing (Genewiz).

### Bacterial strains and plasmids

Supplementary Tables S2 and S3 describe the *E. coli* plasmids and strains, respectively used in this study.

### DNA modification and construction of plasmids containing mutant *dnaA* genes

We employed site-directed mutagenesis to construct plasmids containing different mutant *dnaA* genes (Supplementary Table S1). For this, a pair of external primers (CS4 and CS30) containing NdeI and BamH1 restriction enzyme sites, and internal primers containing bases to introduce mutations in *dnaA* were designed. Two sets of PCR reactions were initiated with each reaction mixture containing Taq DNA polymerase, a pZL411 (38) plasmid (as template DNA) and a pair of external and internal primers. Next, an overlap extension PCR was performed using amplified DNA products obtained in primary reactions (mixed in 1:1 molar ratio) as DNA template and external primers. The amplified product from secondary PCR and plasmid pZL411 were digested with NdeI and BamH1. The digested vector backbone obtained from pZL411 and inserts obtained from PCR amplification were mixed (molar ratio of 1:6) and ligated using T4 DNA ligase at 4°C overnight. Subsequently, the ligation mixtures were used to transform *E. coli* DH5α, and the transformants were screened for insertion of mutated *dnaA* genes. Mutations in *dnaA* were confirmed by DNA sequencing. Mutated *dnaA* genes (Supplementary Table S2) were also cloned under an arabinose-inducible promoter present in pBAD24c. Plasmids pZL606 (39), pRS9 (carrying *dnaA*F76Y), pRS10 (carrying *dnaA*F76A), pRS11 (carrying *dnaA*H136Q), pRS12 (carrying *dnaA*H136A), pRS13 (carrying *dnaA*Q156E) and pRS14 (carrying *dnaA*Q156N) were digested with NdeI and HindIII, and the resulting *dnaA* fragments were ligated to similarly digested vector, pBAD24c, and the transformants characterized as above.

### Protein expression and purification

The expression and purification of 10X-histidine-tagged WT and mutant DnaA (amino acid residues 1–467) proteins were performed as previously described (38), unless stated otherwise. Protein concentrations were determined using Bradford reagent, and purified proteins subsequently stored in small aliquots at −80°C. Proteins examined in SAXS experiments were purified with a minor modification that involved omitting sucrose and including 5% glycerol in buffers used throughout the procedure. Additionally, proteins eluted from Ni-NTA column were concentrated using Amicon Ultra-15/50 centrifugal filter units (Millipore) and subjected to 16/60 Sephacryl-300 HR (GE, Amersham) column chromatography. Pooled fractions containing monomeric DnaA protein were further concentrated to ∼1 mg/ml and the concentration determined using a Nanodrop spectrometer. Nucleotide forms of DnaA were generated by incubating proteins with 1 μM or 0.5 mM ADP or 0.5 mM AMP-PNP. Subsequently, the Apo and the nucleotide forms of proteins were dialyzed against modified HD buffer (50 mM PIPES (piperazine-*N*,*N*′-bis(2-ethanesulfonic acid) pH 6.8, 200 mM ammonium sulfate, 10 mM magnesium acetate, 5% glycerol, 2 mM DTT and 0.1 mM EDTA) in absence or presence of 1 μM or 0.5 mM adenine nucleotides, respectively. The buffers used for dialyzing protein samples were prepared either in H_2_O or 90% D_2_O containing 5% glycerol.

### Small-angle scattering analysis

SAXS experiments were conducted using a Rigaku Bio-SAXS 2000 at 10°C. The instrument software was used to reduce the data to scattering intensity, *I*(*Q*), versus wave vector transfer, *Q* = 4π sin(θ)/λ, where 2θ is the scattering angle, and then to subtract the buffer background. Upon performing Guinier fits (40) to the scattering profiles, the pair distance distribution function, *P*(*r*), was calculated from *I*(*Q*) using the indirect Fourier transform method implemented in the GNOM program (41). The *P*(*r*) function was set to zero for *r* = 0 and *r* = *D*_max_, the maximum linear dimension of the scattering object. *D*_max_ was explored to optimize the *P*(*r*) solution and excellent quality solutions were found in each case. The *P*(*r*) solution to the scattering data yielded the real-space radius of gyration, *R*_g_, and scattering intensity at zero angles, I(0). The SASSIE program (42) was used to generate ensemble models for the entire DnaA oligomer states, ranging from monomer to pentamer. Structures for *E. coli* DnaA domain I (2E0G.pdb) and *A. aeolicus* domains III and IV (2HCB.pdb) were used to create starting models. Domains III and IV from *A*. *aeolicus* (residues 77–399) align with those of *E. coli* (residues 130–467). The coordinates for *E. coli* DnaA domain II (residues 109–129) were generated using the psfgen package within VMD (43), and the structures were minimized within SASSIE (42). Monte Carlo simulations of full-length DnaA were run in SASSIE for monomer through pentamer, allowing domains I and II to move while domains III and IV were held fixed. The trajectories, with 10 000 conformers per oligomer state, were then refined against the Apo DnaA SAXS data to obtain the ∼2000 models with the lowest χ^2^ values for each oligomer. These models were then pooled and input into the GAJOE program of the ensemble optimization method (EOM) (44) to select, using a genetic algorithm, the best 1000 structures as an ensemble average to fit each SAXS curve. Custom Python scripts were written and run to extract the individual oligomer contributions from the EOM fit to scattering curves. The procedure with EOM was repeated multiple times to ensure consistent results. SANS experiments were performed on the extended Q-range small-angle neutron scattering (EQ-SANS, BL-6) beam line at the Spallation Neutron Source (SNS) located at Oak Ridge National Laboratory (ORNL) using a 4 m sample-to-detector distance and two modes: 1) 30 Hz with 2.5-6.1 and 9.4-13.4 Å wavelength bands and 2) 60 Hz with 13-16.1 Å wavelength band. SANS measurements were performed at 10°C in D_2_O buffer solution, and data reduction followed standard procedures using MantidPlot.

### Assay for *oriC-*dependent plasmid replication *in vivo*

To assay p*oriC* replication, *E. coli* EH3827 (*dnaA* null, Kan^R^) cells (45) were first transformed with plasmids containing WT or *dnaA* mutant genes cloned under an arabinose inducible promoter (Supplementary Table S3), and transformants were selected for growth on LB-agar plates supplemented with ampicillin (100 μg/ml). Single colonies were used to inoculate LB medium supplemented with 0.2% arabinose and the above antibiotics, and the cultures were grown at 37°C to an approximate OD_595_ of 0.3 before transforming them with pAL70 (p*oriC*, Cm^R^). Transformants harboring pAL70 were selected for growth on LB-agar plates supplemented with 0.2% arabinose, ampicillin (100 μg/ml) and chloramphenicol (12.5 μg/ml). The number of colonies from biological replicates were recorded and averaged.

### Nucleotide binding assay

Filter retention assays were performed as described earlier (38). Briefly, DnaA protein (∼ 0.15–0.9 μM) was incubated with 1 μM [α-32]-ATP in PP-60 buffer for 15 min at 4°C. To calculate dissociation constants, proteins (∼1.9 μM) were incubated with different concentrations of ^32^P-αATP (0.005–2.5 μM) in PP-60 buffer for 15 min at 4°C. Reaction mixtures were filtered through nitrocellulose membranes presoaked in wash buffer and dried under a lamp. Radioactivity retained on the filter was measured in a liquid scintillation counter.

### DMS footprinting

WT or mutant DnaA proteins (20 and 160 nM, respectively) were incubated with pBS*oriC* and 0.5 mM ATP at 38°C for 7 min in a reaction mixture as described earlier (33). Following dimethyl sulfate and piperidine treatment, samples were processed for primer extension reaction using radio-labeled primers RS4 and SR4, and the extension products were resolved in 6% urea–acrylamide gels (31,32). Dried gels were scanned in the Storm-840 phosphoimager (GE Amersham) to calculate band intensities employing the Image Quant software.

### P1 endonuclease assay

Plasmid p*oriC* (60 fmoles) in 10 μl of footprinting buffer (40 mM HEPES–KOH, pH 7.6, 8 mM MgCl_2_, 30% glycerol, 0.32 mg/ml BSA and 25 ng hydroxyurea (HU) was incubated with nucleotide-bound forms of WT and mutant DnaA proteins at different concentrations (0, 10, 20, 30 nM) for 5 min at 38°C. The mixture was treated with 0.6 units of P1 endonuclease (Sigma) for 10–15 s. The reactions were stopped with stop buffer (2% SDS and 50 mM EDTA) and samples were electrophoresed in 1% agarose gels. Assays were performed in triplicates and images were analyzed by using Image Quant software. A representative scan is shown in the Results.

### Molecular modeling

Structure of *A. aeliocus* DnaA (PDB ID: 1L8Q) was used as a template to guide and construct a model of *E. coli* DnaA (130–369 amino acids) using the SWISS-MODEL server (46). For docking of ATP, we have used the crystal structure of *A. aeolicus* DnaA bound with AMPPCP (PDB ID: 2HCB). The final model was energy minimized using the Amber software (47).

## RESULTS

### Small-angle X-ray scattering (SAXS) suggests that DnaA is present as a monomer-dimer mixture in solution

**Figure 1.**
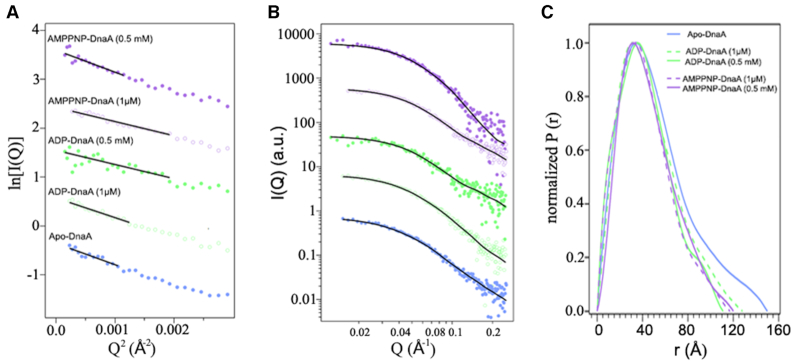
SAXS parameters for different nucleotide forms of *E. coli* DnaA. SAXS profiles (circles) for Apo, ADP and AMP-PNP forms of DnaA calculated using the flexible-oligomer model and the corresponding fits (solid lines) were overlaid on each profile. (**A**) Guinier plots and (**B**) SAXS curves displaced along the logarithmic axis for better visualization. Color assignments for forms of DnaA are same in (A) and (B). (**C**) The distance distribution function *P*(*r*) curves calculated from the SAXS data for the Apo, ADP and AMP-PNP forms of DnaA.

**Figure 2.**
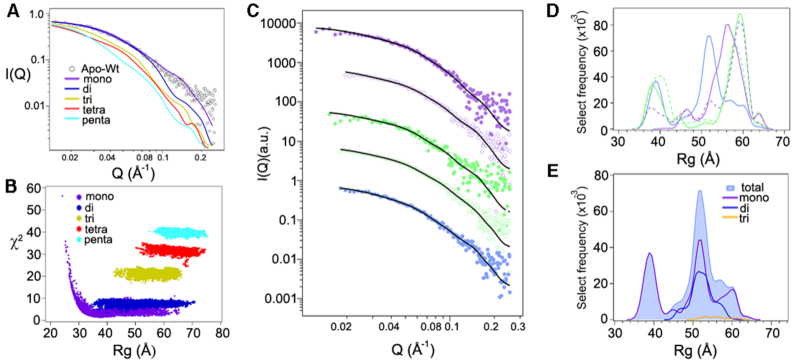
Apo-DnaA is a mixture of different oligomer states. (**A**) SAXS curve for Apo-DnaA (open circles) compared to the lowest discrepancy (χ^2^) values for each oligomeric form, distributed among monomer to pentamer forms (solid lines). (**B**) Comparison of discrepancy (χ^2^) to *R*_g_ values for the best conformers (≤2000 conformations constructed per oligomer) to fit the experimental SAXS data obtained for Apo-DnaA. (**C**) SAXS data (circle) for each oligomeric form present within the Apo-DnaA sample were calculated using flexible-oligomer model and overlaid by corresponding fits (solid lines) on each curve. The curves were shifted in decade increments for better visualization and matched for the color sequences as in Figure 1. (**D**) Histograms of total select models versus *R*_g_ for the different oligomeric DnaA states. The color sequences were matched with (C). (**E**) Histogram of Apo-DnaA total select models versus *R*_g_. The histograms for each contributing oligomer were also plotted for comparison.

SAXS is an extensively used approach to study propensity of proteins in solution to form oligomers (36,37,48). We performed SAXS on nucleotide-free DnaA (apo-DnaA). The initial fits to the SAXS data on Apo-DnaA included Guinier and pair distance distribution fits (Figure 1). All fits were reasonable and indicated the absence of any aggregated species. The resulting fit parameters are shown in Supplementary Table S4. Analysis of the scattering data indicates that DnaA species were slightly larger in size than if it were purely monomer (49), which has a molecular mass of ∼ 52 kDa. A modeling and fitting scheme was implemented to account for a mixture of DnaA oligomeric states in the scattering data, in addition to flexibility within domain II of each subunit (see Materials and Methods for details). Scattering profiles were compared to the calculated scattering from model oligomers, from monomer to pentamer (Figure 2A). Our approach to model both oligomerization and flexibility was similar to earlier scattering studies (36,37). Our results show that a mixture of oligomers was required to appropriately represent the DnaA ensemble state. However, it was apparent from the best fit (lowest χ^2^) structure for each oligomer that the solution structure of apo-DnaA was best described as a monomer, although not completely (χ^2^ = 1.38) (Figure 2B).

By selecting from the pool of oligomers, and accounting for the flexibility of domain II, we were able to achieve an ensemble yielding a lower χ^2^ (= 0.74) in the fit to the apo-DnaA SAXS curve (Figure 2C). The select conformers can be visualized in a histogram of *R*_g_ values (Figure 2D). The contribution of each oligomer to the overall ensemble of apo-DnaA is shown in Figure 2E, where there is considerable overlap in *R*_g_ values for the monomer, dimer and trimer structures, and the generic algorithm did not choose any tetramer or pentamer structure for the best-fit ensemble for this case.

### ATP-stabilizes a DnaA dimer structure in solution

**Figure 3.**
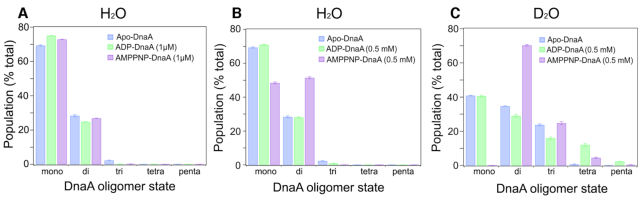
Non-hydrolysable ATP analog, AMP-PNP, promotes dimer formation. The oligomer distributions were determined from the flexible-oligomer model fits to the SAXS data for Apo, ADP and AMP-PNP forms of DnaA. The samples were dialyzed in buffer prepared in H_2_O (**A** and **B**) or D_2_O (**C**). (A) Apo-DnaA or DnaA in the presence of 1 μM ADP and 1 μM AMP-PNP. (B and C) Apo-DnaA or DnaA in the presence of 0.5 mM ADP and 0.5 mM AMP-PNP. Error bars reflect the standard deviation from three separate EOM runs.

In the presence of sub-micromolar concentrations of ADP or ATP, DnaA can bind *oriC* but approximate cellular levels of adenine nucleotides are required to produce replication-competent complexes (33). This led us to compare how adenine nucleotides at micromolar (1 μM) and approximate cellular levels (0.5 mM) influence the assembly state of the initiator protein. To collect SAXS data, we incubated apo-DnaA with 1 μM ADP, 1 μM AMP-PNP, 0.5 mM ADP or 0.5 mM AMP-PNP and dialyzed the mixtures against buffers containing the same amounts of adenine nucleotides. Using the same flexible-oligomer model approach, the best ensemble determined was fitted to ADP- and AMP-PNP-DnaA SAXS profiles (Figure 1A and B). The select frequency versus Rg histograms for all cases are shown in Figure 2D. Compared to the Apo form, the ADP-DnaA histogram shows a slight increase toward larger Rg values. We then analyzed the percent of each oligomer contribution to the fits to examine the shifts in oligomer states more closely (Figure 3A and B). Apo-DnaA was primarily monomer (69.4%) and the rest were dimer (28.4%) and trimer (∼2.2%). The distributions were essentially same in the presence of both 1 μM ADP-DnaA, and 1 μM AMP-PNP-DnaA since majority of the species was monomer (75% and 73%, respectively) followed by dimer (25% and 27%, respectively), and negligible amounts as trimer (0, and 0.1%, respectively) (Figure 3A). The inclusion of 0.5 mM ADP also did not affect the oligomeric distribution since it was 71% monomer, 28% dimer and 0.9% trimer (Figure 3B). In contrast, the addition of a similar amount of AMP-PNP significantly decreased the monomer population to 49% and increased dimer population to 52%. This indicates that the AMP-PNP confers on DnaA a propensity to form higher order structures (Figure 3B).

Often, the presence of different assembly states in the mixture is attributed to the status of protein hydration due to the solvent. To address this, we performed SAXS for the protein samples in D_2_O, which is known to favor the assembly, and sometimes aggregation of protein by changing its hydration status (50). DnaA samples were dialyzed against buffers prepared in D_2_O in the presence of 0.5 mM ADP and 0.5 mM AMP-PNP, and oligomer distributions were estimated. The same flexible model approach was taken to determine the best ensemble fits to the ADP-DnaA and AMP-PNP-DnaA SAXS profiles (Supplementary Figures S1 and S2 & Table S4) and select distribution of oligomeric states are shown in Figure 3C. All states (Apo, 0.5 mM ADP- and AMP-PNP-bound) showed a shift toward larger oligomers in the presence of D_2_O buffer that now include besides dimers and trimers some tetramers and pentamers. Most notably, DnaA-AMP-PNP was highly enriched in dimers (∼70%), while the monomers were almost totally absent. To confirm our results, we performed Small Angle Neutron Scattering (SANS) on these samples (Supplementary Figure S3 and Table S5), which further indicated that only DnaA-AMP-PNP favors dimerization.

To test further the influence of adenine nucleotides on the assembly state of DnaA protein in solution, we used an amine-specific cross-linking agent, DTSSP (3,3′-dithiobis(sulfosuccinimidyl propionate) (51). DnaA in the presence of 0.5 mM ADP, AMP-PNP, or ATP was cross-linked with DTSSP, and the samples were analyzed by SDS-PAGE and subsequently by western blotting using an anti-DnaA antibody. Compared to apo-DnaA or DnaA in the presence of 0.5 mM ADP, there was a significant increase in dimer formation for DnaA in the presence of 0.5 mM AMP-PNP or 0.5 mM ATP (Supplementary Figure S4). These results are fully consistent with the SAXS and SANS data, which indicated the influence of ATP in stabilization of DnaA dimers in solution.

### Changes at the His136 residue make chromosomal replication initiation defective *in vivo*

As indicated by SAXS, SANS and cross-linking experiments, the stabilization of DnaA-AMP-PNP or DnaA-ATP dimers occur at an adenine nucleotide concentration needed to facilitate conformational change between ADP-DnaA versus ATP-DnaA (33). This change is required to convert DnaA-*oriC* complexes to an initiation-competent state. We have previously identified several amino acid residues, such as Phe-76, His136 and Gln-156, where these conformational changes might occur (33). Multiple alignments using Clustal X 2.1 software (52) of protein sequences from various members of γ- proteobacteria (Supplementary Figure S5) identified the conserved nature of these amino acid residues. These observations led us to investigate the importance of these residues in the initiator function of DnaA.

We performed site-directed mutagenesis to construct mutant *dnaA* genes. Wild-type and mutant *dnaA* genes were cloned in pBAD24c vector (Supplementary Table S2) so that *dnaA* expression could be controlled by adding arabinose to the growth media (53). We used the *E. coli* EH3827(Δ*dnaA*) strain (45), which does not require DnaA for growth since initiation of chromosomal replication occurs from an integrated miniR1 plasmid (pKN500), which does not require DnaA. The initiator activity of the plasmid-borne *dnaA* genes was assessed by measuring cell growth that is dependent on the replication of an *oriC* plasmid, pAL70 (p*oriC*). EH3287 cells expressing WT or mutant DnaA proteins (Table 1) were made competent and transformed with p*oriC*. The cells expressing WT or mutant DnaA proteins when plated (after serial dilution) on LB-Agar plates supplemented with ampicillin and 0.2% arabinose, but without chloramphenicol showed no apparent differences in the number of colony-forming units (Table 1). The inclusion of chloramphenicol did not show any growth for cells containing only pBAD24c vector, as expected from non-functionality of p*oriC* in the absence of DnaA (Table 1). While cells expressing WT, DnaA(F76Y), DnaA(F76A), DnaA(Q156E) and DnaA(Q156N) proteins, showed reduced number of colony-forming units when compared to no chloramphenicol controls (Table 1). The colony-forming units for cells expressing DnaA(H136A) and DnaA(H136Q) were at least an additional four orders of magnitude lower. These results are in agreement with a previous report that DnaA(H136A) is unable to initiate replication from *oriC in vivo* (54). Here we have also substituted His136 residue with Gln, as their partially conserved nature is thought to help maintain the three-dimensional structure of native proteins. Like DnaA(H136A), DnaA(H136Q) also did not produce any colonies on LB-Agar plates supplemented with ampicillin, chloramphenicol and arabinose, further arguing that DnaA harboring mutations at His136 can make the protein non-functional in initiation of replication from *oriC*.

**Table 1.** Substitutions at H136 does not support initiation of replication *in vivo*: *E. coli* EH3827 cells were transformed with pZL606 and pRS9-pRS14 plasmids and the transformants were grown for 10–12 generations. Proteins were expressed by adding 0.2% arabinose and growing the cultures for an additional 90 min. Protein lysates were prepared and the protein amounts in the lysate were estimated using the Bradford assay by loading equal amounts of total protein in each lane (lanes 1–9). Samples were analyzed by SDS-PAGE and after transfer to the PVDF membranes and western blotting. The blots were probed with polyclonal anti-DNA antibody and reprobed with anti-RNAP antibody (treated as a loading control). A representative scan is presented here to show the expression level of DnaA. Numbers indicate the relatives ratios of DnaA protein to total protein in each lane. Next, cells containing different *dnaA* alleles under arabinose-inducible promoter were made competent and transformed with plasmid containing *oriC*. The transformed cells were plated at LB-Agar plates supplemented with 0.2% arabinose and appropriate antibiotics (see material and methods). Data represent average values from three biological replicates

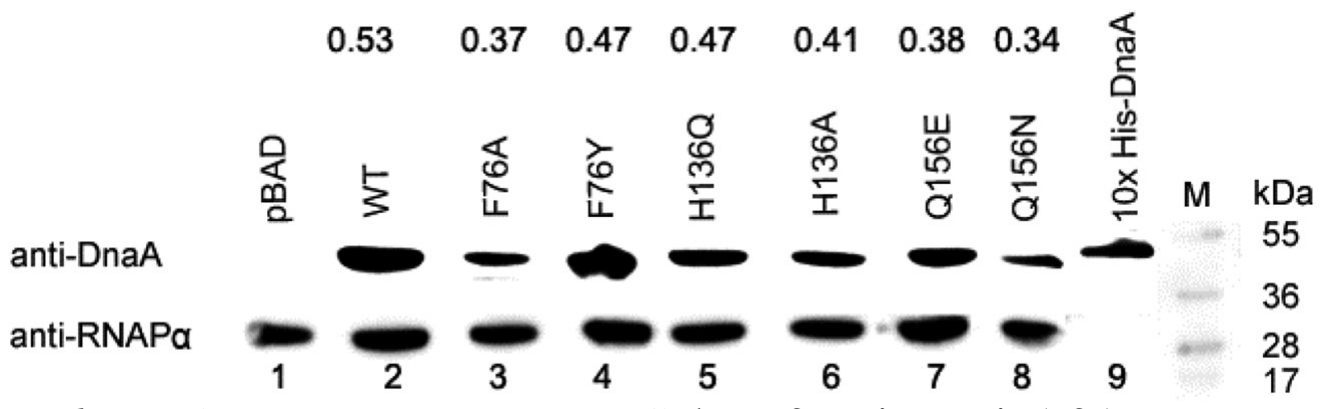		
*E. coli* EH3827	Colony forming unit (cfu)/μg	
With	Ampicillin (0.2% arabinose)	Ampicillin + chloramphenicol (0.2% arabinose)
pBAD	1.9 × 10^7^	<1
pZL606(*dnaA)*	*2*.0 × 10^7^	2.5 × 10^4^
pRS9(*dnaA*F76Y)	4.4 × 10^7^	1.6 × 10^4^
pRS10(*dnaA*F76A)	2.8 × 10^7^	3.4 × 10^4^
pRS11(*dnaA*H136Q)	3.4 × 10^7^	<1
pRS12(*dnaA*H136A)	3.0 × 10^7^	<1
pRS13(*dnaA*Q156E)	2.4 × 10^7^	3.4 × 10^4^
pRS14(*dnaA*Q156N)	2.5 × 10^7^	3.4 × 10^4^

### DnaA(H136Q) retains ATP binding activity

Purified histidine-tagged WT and mutant DnaA proteins (Supplementary Figure S6B) were used to assess their ATP-binding properties in a filter retention assay (Table 2). The DnaA: ATP binding stoichiometry for WT DnaA (0.29 ± 0.06, Table 2) was found to be similar to those published earlier (54,55). The stoichiometries for the DnaA mutants with Phe76 substituted with Ala or Tyr were 0.36 ± 0.07 or 0.22 ± 0.03, and for His136 substituted with Gln was 0.19 ± 0.01, and for Gln156 substituted with Asp was 0.33 ± 0.05. Thus, the ATP to DnaA stoichiometry did not change drastically in any of the above mutants (Table 2). The substitution of His136 with Ala and Gln156 with Asn change, however, reduced the stoichiometry to 0.08 ± 0.03 and to 0.04 ± 0.04, respectively. However, binding by wildtype or mutant DnaA proteins (1.9 μM) to ^32^P-αATP (in the range between 0.005 and 2.5 μM) showed typical binding curves with dissociation constants (*K*_D_) in sub-micromolar range in all cases (Table 2). Our results obtained with DnaA(H136A) mutant, which has lower ATP binding stoichiometry (Table 2), but dissociation constants (*K*_D_) similar to wildtype are in agreement with a recently published report (54).

**Table 2.** ATP binding affinity of WT and mutant DnaA proteins: DnaA proteins were incubated with radioactive ATP and nucleotide binding was measured by filter retention assay (see Materials and Methods). Mean (±SD) values represents data from three independent set of experiments

Amino acid change	ATP binding stoichiometry (fmoles ATP/fmoles protein)	*K*_D_ (ATP binding) (μM)
WT	0.30 ± 0.03	0.11 ± 0.03
F76Y	0.22 ± 0.03	0.14 ± 0.03
F76A	0.36 ± 0.07	0.12 ± 0.03
H136Q	0.19 ± 0.01	0.10 ± 0.02
H136A	0.08 ± 0.03	0.14 ± 0.04
Q156E	0.33 ± 0.05	0.10 ± 0.02
Q156N	0.04 ± 0.01	0.10 ± 0.02

### DnaA(H136Q) fails to convert DnaA-*ori*C complexes to a replication-competent state

**Figure 4.**
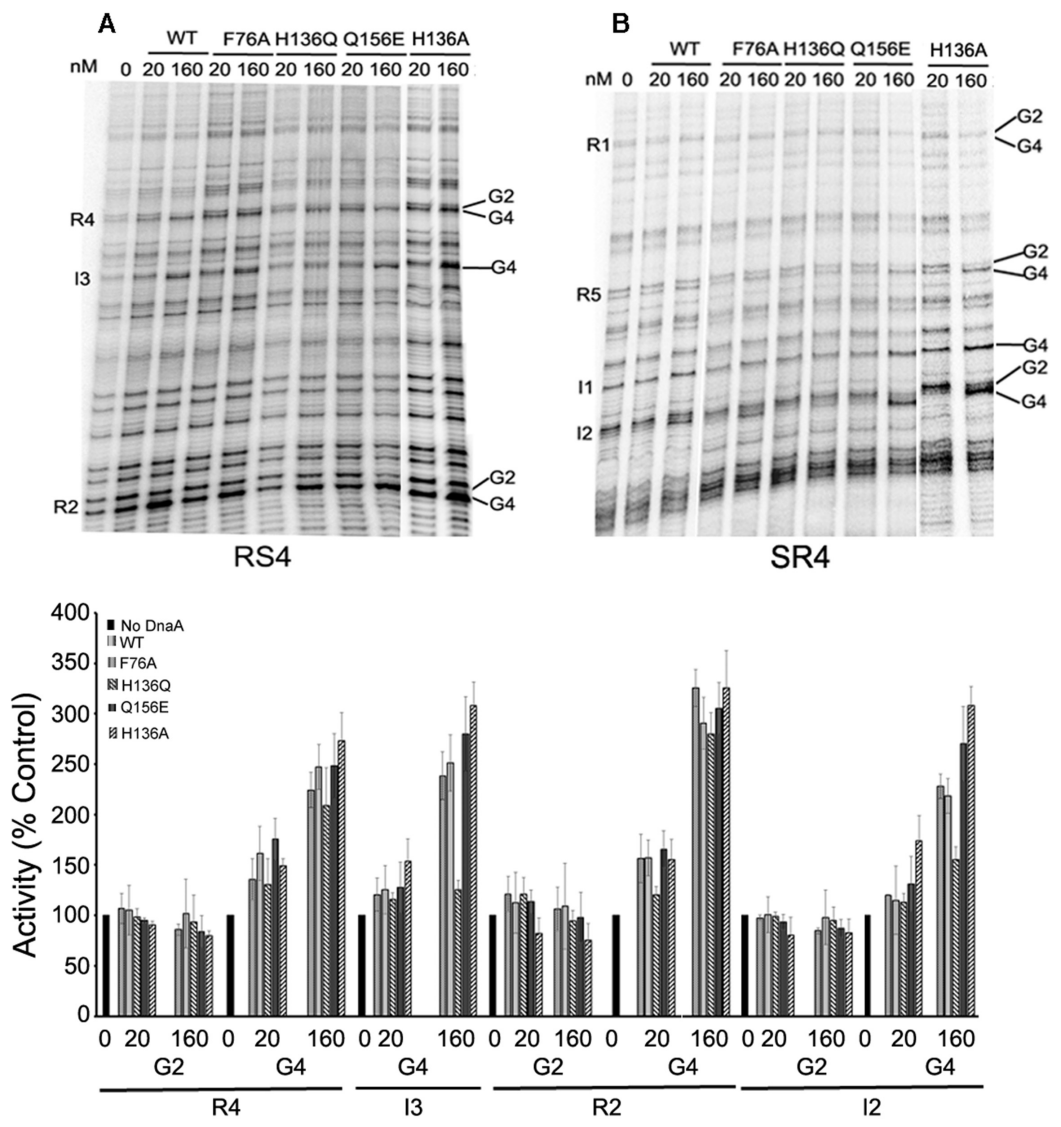
DnaA(H136Q) fails to bind with low-affinity sites present within *oriC*. *In vitro* dimethyl sulfate footprint analysis was performed with WT and mutant DnaA proteins (20 nM and 160 nM) in the presence of 0.5 mM ATP to assess their occupation of low and high-affinity sites present within *oriC*. (Top) Two primers (**A**) RS4 and (**B**) SR4 were used to carry out primer extension to monitor changes in intensities of G2 and G4 guanines present within DnaA recognition sites. (Bottom). The band intensities of modified guanines present at R2 and R4 (representing high-affinity sites), and I2 and I3 (representing low-affinity sites) were plotted relative to respective intensities within the no-protein lanes.

In order to investigate the effect of DnaA with the changed residues to produce replication-competent nucleoprotein complexes at *oriC*, the complexes were probed with dimethyl sulfate (DMS). DMS changes the methylation pattern particularly at guanines, G2 and/or G4, present within both high- and low-affinity DnaA binding sites in *oriC* (5,31–33). Reactions were initiated by adding purified DnaA, DnaA(F76A), DnaA(H136Q) and DnaA(Q156E) to pBS*oriC* in the presence of 0.5 mM ATP, as described previously (33). Changes in the methylation pattern of guanines were monitored at DnaA binding sites R2 and R4 (representing high-affinity sites) as well as I2 and I3 (representing low-affinity sites) (Figure 4, top panel). The changes in band intensities reflecting the extent of DMS reactivity were calculated relative to the no protein control (Figure 4, bottom panel). In the presence of 20 nM and 160 nM proteins, band intensity at G4 within R2 site (Figure 4) increased 1.6–3.3-fold for WT DnaA, 1.6–2.9-fold for DnaA(F76A), 1.2–2.8-fold for DnaA(H136Q) and 1.7–3.1 for DnaA(Q156E). The increase at G4 within R4 was 1.4–2.2-fold for WT DnaA, 1.6–2.5-fold for DnaA(F76A), 1.3–2.1-fold for DnaA(H136Q) and 1.8–2.5 for DnaA(Q156E) (Figure 4, bottom panel). These results indicate that the mutant proteins are competent in binding *oriC* high-affinity sites, similarly to the WT.

**Figure 5.**
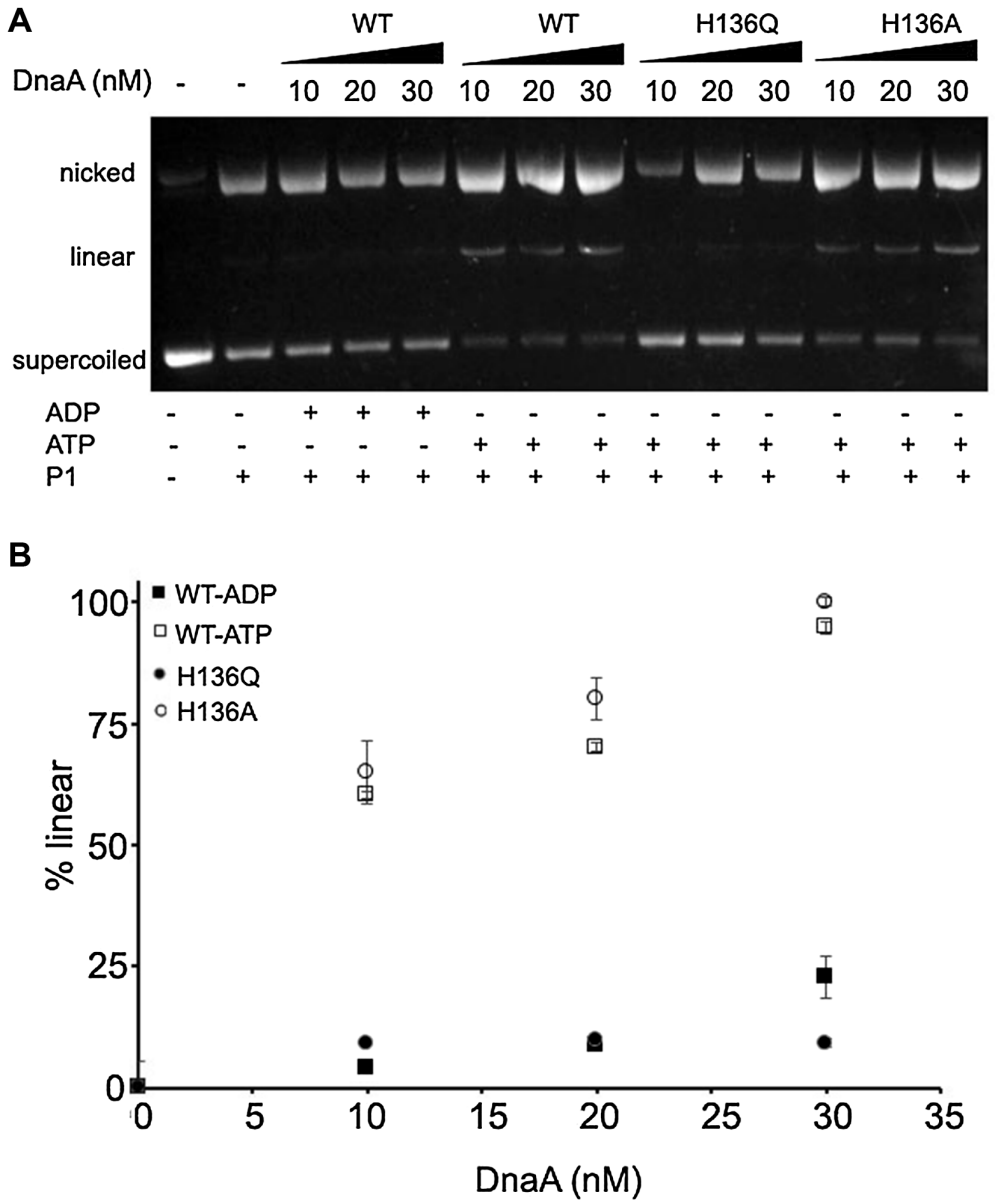
DnaA(H136Q) is defective in opening the strands of *oriC*. Single-strand specific endonuclease P1 was used to measure ability of WT and mutant DnaA proteins to open the DNA duplex. The indicated amounts (0, 10, 20, 30 nM) of ADP-DnaA (•), ATP-DnaA (○), ATP-DnaA(H136Q) (▪), and ATP-DnaA(H136A) (□) were incubated with 60 fmol of supercoiled pAL70 (p*oriC*) plasmid for 5 min at 38°C. Samples were treated with P1 endonuclease (0.6 units) for 10–15 s, and reactions were stopped by adding a stop solution and immediate transfer of the samples to ice. Different forms of plasmid DNA were separated by electrophoresis followed by ethidium bromide staining. The relative percentage of linear form was quantified using Image Quant software. Data represents the mean (± SD) from three independent sets of experiments.

Occupation of the low-affinity sites (represented by I2 and I3) determines whether *oriC* has been converted to a replication-competent state (34–37). WT DnaA increased the methylation of G4 1.2–2.3-fold in I2 and 1.2–2.4 fold in I3, which is indicative of successful conversion of the origin to the replication-competent state (Figure 4, bottom panel). Both DnaA(F76A) and DnaA(Q156E) showed an increase in the methylation pattern similar to that for WT protein (Figure 4, *top panel*). For example, DnaA(F76A) increased methylation 1.1–2.2 fold at I2 and 1.3–2.5-fold at I3. Similarly, DnaA(Q156E) increased methylation 1.3–2.7-fold at I2 and 1.3–2.8-fold at I3 (Figure 4, *bottom panel*). In contrast, band intensities with DnaA(H136Q) was comparable to those in the no protein control lane, indicating only marginal binding to I2 and no binding to I3 (Figure 5, see top and bottom panel). These results demonstrate the importance of His136 in mediating the conversion of DnaA-*oriC* complexes to a replication-competent state.

A recent study indicates that DnaA(H136A) enables the unwinding of DNA duplex but fails to initiate replication both *in vitro* and *in vivo* due to a defect in loading of DnaB helicase at single strands (54). We examined the ability of DnaA(H136A) to occupy low-affinity sites present within *oriC*. Contrary to DnaA(H136Q), DnaA(H136A) increases methylation 1.7–3.0-fold at I2 and 1.5–3.0-fold at I3 (Figure 4, bottom panel), which further support its ability to unwind duplex DNA. Perhaps not surprisingly, the behavior of a DnaA mutant appears to depend upon the nature of the amino acid substitution aside from the location of the substitution in the primary sequence of the mutants. To confirm this, we have performed P1 endonuclease assay, where an increase in the linear form of *oriC* DNA indicates the ability of DnaA protein to unwind duplex DNA. Our results suggest that unlike DnaA(H136A) that supported duplex unwinding (54), DnaA(H136Q) was unable to unwind the DNA duplex, explaining why it is inactive as an initiator in *in vitro* replication (Figure 5).

We have previously shown that upon limited proteolysis with trypsin, ADP-DnaA but not ATP-DnaA generates relatively stable proteolytic fragments, particularly a 33 kDa DnaA fragment containing amino acid residues His136-Arg432 (33). The concentrations of the adenine nucleotide needed to induce conformational changes as detected by proteolytic susceptibilities of DnaA, coincided with the conversion of inactive DnaA-*oriC* complexes to replication-efficient DnaA-*oriC* complexes (33). To further examine why DnaA(H136A), but not DnaA(H136Q), can unwound double stranded DNA, we subjected the two forms to limited proteolytic digestion. Our results indicate that upon limited proteolysis only DnaA(H136Q), even in the presence of 0.5 mM ATP generates the stable 33 kDa DnaA fragment (Supplementary Figure S7), indicating that it is in the inactive ADP-DnaA form, unlike DnaA(H136A), which appears to be in the active ATP-DnaA form. These results suggest that with the substitution of His136 with Gln, DnaA may not be able to undergo the nucleotide-dependent conformational change required for the formation of replication-competent DnaA-*oriC* complexes.

### Nucleotide-induced conformational change at DnaA(H136Q) causes rearrangement in the ATP binding pocket that diminishes stable DnaA dimerization

**Figure 6.**
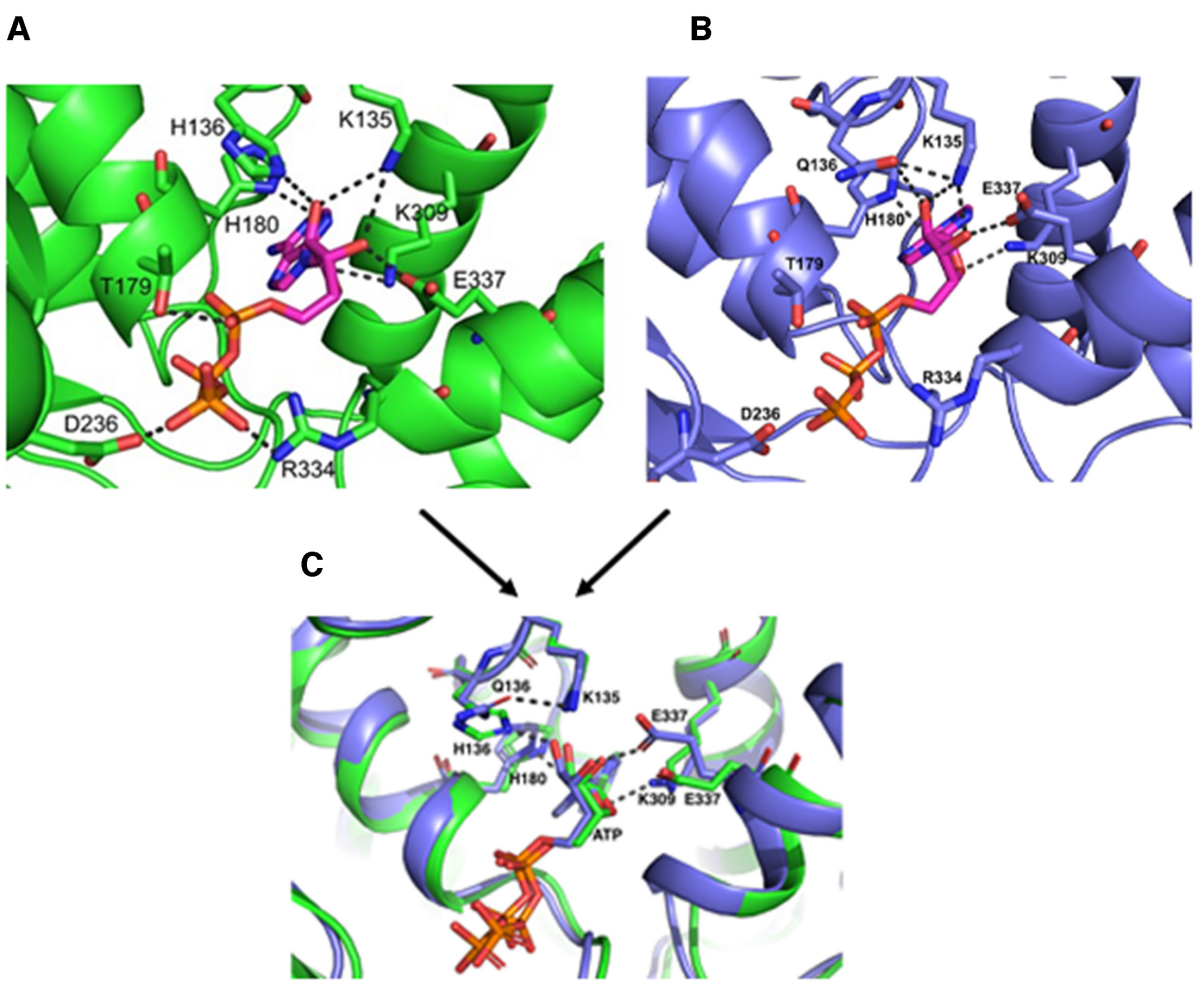
His136 residue of DnaA participates in ATP binding. Homology model of *E. coli* DnaA (amino acid residues 130–369) was constructed upon an *A. aeolicus* DnaA structure (PDB ID: 1L8Q) using the SWISS-MODEL server. The crystal structure of *A. aeolicus* DnaA bound to AMPPCP (PDB ID: 2HCB) was used to perform docking of ATP into the active site of *E. coli* DnaA and the final model was subsequently energy minimized using Amber software. The ATP binding pocket in DnaA WT (**A**) and in DnaA Gln136 mutant (**B**). The structures in (A) and (B) are superimposed in (**C**).

To obtain insights into the structural role played by His136, we decided to construct a homology model of *E. coli* DnaA (amino acid residue 136–367) using *A. aeliocus* truncated DnaA structure containing AMP- PCP in its nucleotide-binding pocket (23). Although the homology modelling studies were based on the available structures obtained at ATP concentrations lower than the physiological concentration, these studies served us well in pointing out the structural rearrangement in the nucleotide-binding pocket upon replacement of His136 with Gln. Our modeled structure suggests that His180 and Lys309 form hydrogen bonds with the adenine base of ATP, and Lys135, Lys309 and Glu337 form hydrogen bonds with hydroxyl oxygen present within ribose sugars at position C2 and C3 (Figure 6A). In addition, hydrogen bonds between amino acid residues Thr179, Asp236 and Arg334 with the phosphate groups of ATP were observed. These interactions might stabilize the binding of ATP with DnaA protein (Figure 6A). The modeled structure also indicates the presence of a hydrogen bond between His136 and hydroxyl oxygen of ribose carbon at position C2 (Figure 6A). Moreover, the nitrogen present within imidazole ring of His136 makes a hydrogen bond with the backbone oxygen of His180 (Figure 6A).

Further, we found that substitution of His136 with Gln causes noticeable re-arrangements in the nucleotide-binding pocket of DnaA protein. The amide group present in Gln136 generates a favorable ionic interaction with the backbone oxygen of His180 in addition to an additional salt-bridge with Lys135 (Figure 6B). Moreover, Glu337 and Lys309 make new ionic interactions with ATP’s sugar moiety (Figure 6B), which may play role in maintaining the stable binding of ATP within nucleotide binding pocket of DnaA (as shown in Table 2). However, the absence of the imidazole group abolishes the hydrogen bond interaction with the hydroxyl oxygen of ribose (Figure 6B).

**Figure 7.**
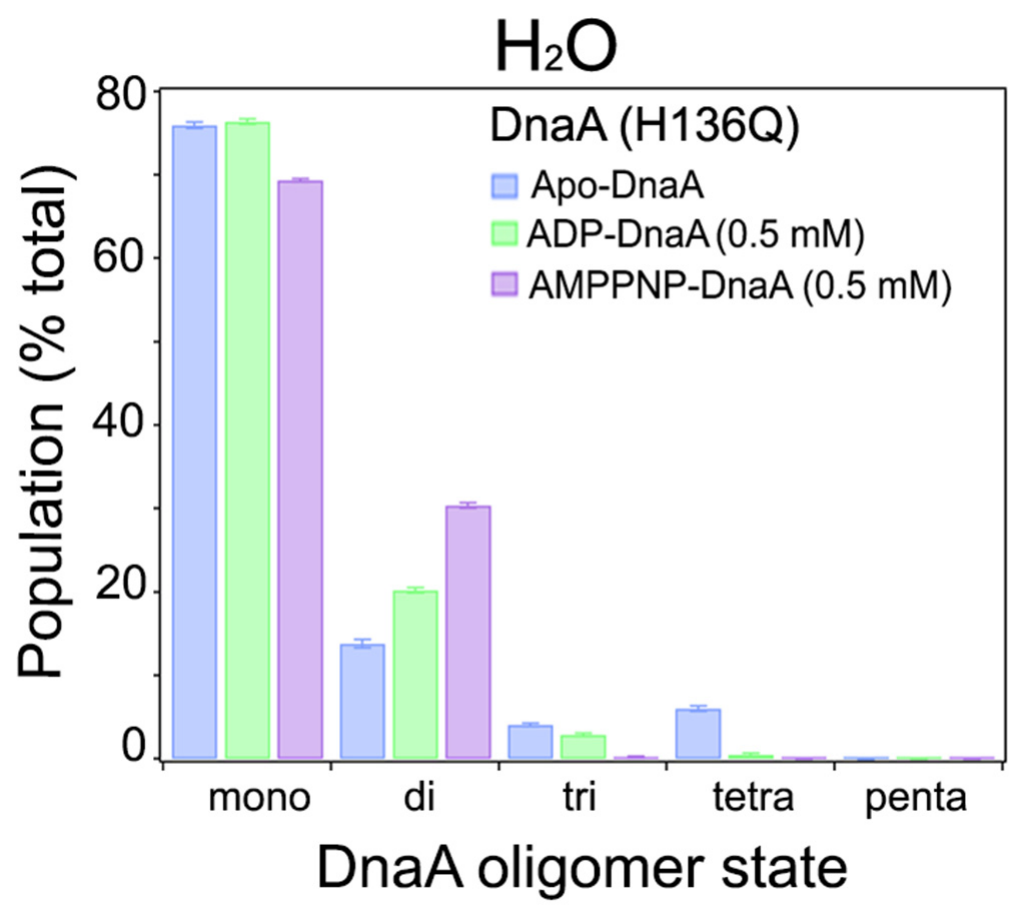
H136Q change interferes with nucleotide-induced dimer formation. Similar to WT (Figure 3), SAXS was carried out to determine the percentage of each oligomeric state of DnaA(H136Q) in the absence or presence ADP (0.5 mM) and AMPNP (0.5 mM). The oligomer distributions were determined with the flexible-oligomer model after fits to the SAXS data. Error bars reflect the standard deviation from three separate EOM runs.

The facts that (i) ATP induces favorable formation of DnaA dimers (Figure 3, panels A–C), (ii) DnaA(H136Q) is compromised for initiation at *oriC in vivo* (Table 1), (iii) DnaA(H136Q) abrogates the extension of DnaA assembly at the chromosomal origin (Figure 4) and (iv) substitution of His136 with Gln causes rearrangements within the ATP binding pocket of DnaA (Figure 6B) prompted us to investigate the oligomer state of DnaA(H136Q). With SAXS and our flexible-oligomer model approach (Supplementary Figure S8 and Table S6), oligomer histograms were calculated for the different states of DnaA(H136Q) (Figure 7). Both the ADP and AMP-PNP bound forms of DnaA(H136Q) predominantly stayed as monomer (Figure 7). Further, in DTSSP (3,3′-dithiobis(sulfosuccinimidyl propionate) crosslinking assay, the AMP-PNP form of DnaA(H136A), but not DnaA(H136Q) showed an increase in the formation of higher order oligomer structures (Supplementary Figure S9). Together, our results demonstrate that ATP-mediated conformational transitions at His136 enable DnaA to form stable dimers, which is essential for the formation of replication-competent complexes.

## DISCUSSION

The members of the AAA+ protein family form higher order oligomeric structures, either without requiring nucleotide binding or in a nucleotide-binding dependent manner. In these higher order complexes, the protein subunits remain arranged in a head-to-tail manner to form primarily a hexameric closed-ring structure, such as in ClpB ATPase (56) and DnaB_6_–DnaC_6_ helicase–helicase loader complexes (57). DnaA, a member of the AAA+ protein family, oligomerizes at *oriC* in a nucleotide-dependent manner to initiate the process of replication (1–4). However, although the association state of DnaA in solution remains unknown, it is believed to be an important factor in the generation of replication-competent DnaA-*oriC* complexes. Another enigmatic feature is the requirement for physiological concentration of ATP for replication initiation when DnaA can bind specifically to the origin DNA at several order of magnitude lower concentration of ATP (28). Here we have attempted to address these outstanding questions on DnaA oligomerization in relation to varying ATP concentration.

High-resolution X-ray crystal structures of domains III-IV of *A. aeliocus* ADP-DnaA show the protein to be primarily monomeric (22) and AMPPCP-DnaA to be primarily tetrameric (23). So far, these studies serve as the only source of DnaA structure that suggest nucleotide-dependent distinct DnaA assembly. In addition, analysis of *E. coli* DnaA domain I by NMR (16) and DnaA domain IV in complex with a DnaA box by X-ray crystallography (27) have provided some valuable insights about DnaA oligomerization and DnaA-*oriC* complex formation. Several approaches have suggested that some 5–20 molecules of DnaA protein can accumulate at a chromosomal origin, although the exact number remains debatable (1,2,28–30). Also not clear is the nature of the nucleotide-dependent DnaA oligomers at *oriC*. Glutaraldehyde crosslinking indicated a varying influence of ADP versus ATP on the oligomeric state of DnaA (35) but these experiments did not suggest the nature of DnaA protein required for origin remodeling to a replication-competent state.

SAXS is a powerful approach to determine the propensity of a protein to form oligomers in solution, whether or not disordered regions are present in the protein (36,37). We have applied SAXS to assess the oligomerization state of full-length *E. coli* DnaA and the influence of adenine nucleotides on this process. SAXS revealed that Apo-DnaA remains primarily as monomer, and the distribution of assembly states is not affected by the inclusion of micromolar (1 μM) amounts of ADP or AMP-PNP (Figure 3A). SAXS analysis of DnaA in the presence of near physiological concentrations of ADP also showed no change in the oligomeric state of DnaA (Figure 3B). These results highlight that the low concentrations of both ADP and ATP or cellular levels of ADP cannot generate a functional DnaA state. These results are in line with the structural analysis indicating that bound ADP causes stearic hindrance between adjacent DnaA monomers (22). In contrast, millimolar concentrations of AMP-PNP comparable to the physiological levels of ATP switches DnaA primarily into the dimer form (Figure 3B and C), indicating that dimers are the functional form of DnaA. Grimwade *et al.* have recently suggested the possibility that the formation of stable DnaA oligomer longer than dimers is not essential for a mutant *oriC* activation, which has lost its preference for ATP-DnaA (51).

We have previously shown that DnaA may undergo a conformational change at a high (∼physiological) ATP concentration that enables the protein to interact with low affinity sites on duplex *oriC* DNA (33). Our study indicated several specific sites present within the DnaA AAA+ domain where ATP-induced conformational changes may occur. Analyses of initiation characteristics of DnaA mutants suggested the importance of DnaAHis136 for the formation of replication-competent complexes *in vitro* (Table 2) and initiation of DNA synthesis at *oriC in vivo* (Table 1). A recent report from Sakiyama *et al.* also showed the inability of DnaA(H136A) to initiate replication at *oriC* in agreement with our *in vivo* results (54). Of note, that although DnaA(H136A) is able to form replication-competent complexes at *oriC in vitro*, its inability to allow replication initiation *in vivo* is likely to be due to its inability to load DnaB helicase on unwound *oriC* (54). The initiation defect of DnaA(H136Q) is easily rationalized from its defect in dimer formation as well as in replication-competent origin complex formation. The Gln136 mutant although can bind ATP, appears to be particularly defective in responding to high ATP, required for oligomerization and replication competence. Therefore, the ability of DnaA(H136Q) to bind ATP and yet its defect in dimerization indicate that the requirements of the two processes are not identical.

The limited proteolysis data indicate that the rearrangements occurring in the nucleotide-binding pocket of DnaA that includes Gln136 are not adequate for DnaA assembly and its replication competence. It is noteworthy that protonation-deprotonation of amino acid residues in proteins are considered as posttranslational modifications, which can drive dynamic changes in protein conformation and related protein functions (58). There is a strong possibility that a partial negative charge developed on the reactive oxygen of ATP during the transition state is stabilized by the positive charge of protonated imidazole ring. The removal of imidazole *via* substitution of His to Gln disrupts the proton relay. Contrary to this, substitution with Ala results in the loss of hydrogen bonds, but this would allow water to occupy the vacated space so that transition state is solvated, and water could function in the proton relay (59). Taking this into consideration, we hypothesize that the protonated state of His136 determines promotion of DnaA to the dimer state in solution. Moreover, it is possible that the His136 residue is used at both an early stage in duplex DNA binding and a later stage of loading DNA helicase on single-strands. Together these results emphasize the importance of the highly conserved His136 residue in the initiator function of DnaA.

Despite extensive studies, many aspects of the initiation step of DNA replication in *E. coli* remain unknown, such as why ATP is required in high concentrations for the initiation process? We show that one reason for this requirement is in DnaA oligomerization. We do not know whether ATP at high concentration can bind to more sites than the one in the AAA+ domain. Moreover, His136 is present within the N-linker region flanking the flexible linker domain II, which is known to properly align well-structured domains, I and III (20). Any role of higher ATP amount in maintaining the function of the domain II will be worth investigating.

## Supplementary Material

gkz939_Supplemental_File
