# Supplementary material for: A nucleotide-dependent oligomerization of the Escherichia coli replication initiator DnaA requires residue His136 for remodeling of the chromosomal origin
Source: Nucleic Acids Res. 2019 Oct 29;48(1):200–11. doi: 10.1093/nar/gkz939 (PMC7145717; doi:10.1093/nar/gkz939)
Supplement: gkz939_Supplemental_File [file gkz939_supplemental_file.pdf]

Supplemental Information

Supplementary Figure S1. (A) SAXS curve for Apo-DnaA in D_2O (open circles) compared to the lowest discrepancy (χ^2) values for each oligomeric form (solid lines), distributed among monomer to pentamer. (B) Comparison of discrepancy (χ^2) to R_g values for selecting the conformers (≤ 2000 conformations constructed per oligomer) that best fit the experimental SAXS data obtained for Apo-DnaA in D_2O.

Supplementary Figure S2. (A) SAXS profiles (circles) for Apo, ADP and AMP-PNP form of DnaA in D_2O calculated using the flexible-oligomer model and the corresponding fits (solid lines) overlaid to each curve. Curves were shifted in decade increments for better visualization. (B) Histograms of total select models vs. R_g for the Apo, ADP and AMP-PNP forms of DnaA in D_2O (C) Histogram of Apo-DnaA total select models vs. R_g. The histograms for each contributing oligomer were also plotted for comparison. (D) Guinier plots (experimental data shown as circles) and the corresponding fits (shown by solid lines) for Apo, ADP and AMP-PNP forms of DnaA in D_2O. The curves were appropriately displaced along the logarithmic axis for better visualization. (E) Pair distribution, $P(r)$, curves calculated from the SAXS data for Apo, ADP and AMP-PNP forms of DnaA in D_2O.

Supplementary Figure S3. (A) SANS profiles (circles) for Apo, ADP and AMP-PNP form of DnaA in D_2O (93%) calculated using the flexible-oligomer model and the corresponding fits (solid lines) overlaid on each curve. Curves were shifted in decade increments for better visualization. (B) Guinier plot and the corresponding fits (shown by solid line) for Apo, ADP and AMP-PNP forms of DnaA in D_2O. (C) Pair distribution, $P(r)$, curves calculated from the SAXS data for Apo, ADP and AMP-PNP forms of DnaA in D_2O.

Supplementary Figure S4. Nucleotide status influences DnaA oligomerization. Reaction mixtures containing 100 nM apo-DnaA protein or DnaA bound to 0.5 mM ADP, 0.5 mM AMP-PNP, 0.5 mM ATP were incubated with cross-linking reagent, DTSSP (3,3'-dithiobis(sulfosuccinimidyl propionate) for 3 min at 38 ºC. Reactions were terminated by the addition of lysine (50 mM), and after adding loading buffer, the samples were resolved on 8% SDS PAGE gels. The proteins after transfer to nitrocellulose membranes were probed with anti-DnaA antibody. Assays were performed in triplicates with a representative gel shown in the Figure. M indicates molecular weight markers.

Supplementary Figure S5. Sequence alignment of multiple DnaA sequences from Gram-negative bacteria was carried out using ClustalX 2.1 program. The conserved regions such as Walker A and Walker B boxes (colored in green) and sensor I, sensor II (colored in yellow) present within AAA+ domain were indicated. The amino acid residues in *E. coli* DnaA predicted to be involved in nucleotide-dependent conformational changes (colored in red) are conserved in different DnaA homologs.

Supplementary Figure S6. (A) Schematic representation of DnaA domains and amino acids associated with conformational changes induced with cellular levels of ATP. (B) Coomassie brilliant blue staining of recombinant histidine-tagged wild-type DnaA and mutant proteins resolved by 15% SDS-PAGE (each lane is loaded with 5 µg). M indicates molecular weight markers. Numbers (*top*) indicate the relative amounts of mutant: wildtype DnaA protein in each lane (2-8).

Supplementary Figure 7. Partial proteolysis reveals conformational changes between DnaA(H136A) and DnaA(H136Q). Nucleotide-free and ATP forms of the two mutant proteins (1.5 µM) were subjected to proteolysis by trypsin for 30 min at 30 ºC in the absence or presence

of 0.5 mM ATP. Reactions were stopped by addition of phenylmethylsulfonyl fluoride (50 mM PMSF) and sample buffer. Samples were heated for 5 min and analyzed by 15% SDS PAGE. R1 indicates the region showing proteolytic fragments with different sensitivity to trypsin. T3 indicates the 33 kDa proteolytic fragment carrying residues His136-Arg432.

Supplementary Figure S8. (A and B) SAXS profiles (circles) for Apo, ADP and AMP-PNP forms of DnaA(H136Q) calculated using the flexible-oligomer model and the corresponding fits (solid lines) were overlaid on each profile. (A) Guinier plots (B) SAXS curves with pair distribution, *P(r)* were appropriately displaced along the logarithmic axis for better visualization. (C) The distance distribution function *P*(*r*) curves calculated from the SAXS data for the Apo, ADP and AMP-PNP form of DnaA(H136Q). (D) Histograms of total select models vs. R_g values for DnaA(H136Q).

Supplementary Figure S9. Deficiency of DnaA(H136Q) in ATP-induced oligomerization. Reaction mixtures containing 100 nM apo-DnaA(H136A) or apo-DnaA(H136Q) in absence or presence of 0.5 mM ADP or 0.5 mM AMP-PNP were incubated with the cross-linking reagent, DTSSP (3,3'-dithiobis(sulfosuccinimidyl propionate), for 3 min at 38 ºC. Reactions were terminated by the addition of lysine (50 mM), and after the addition of loading buffer, the samples were resolved by 8% SDS PAGE. Proteins were transferred to nitrocellulose membranes, which were then probed with anti-DnaA antiserum. Assays were performed in triplicates with a representative gel shown in the Figure. kDa indicates the size of molecular weight markers.

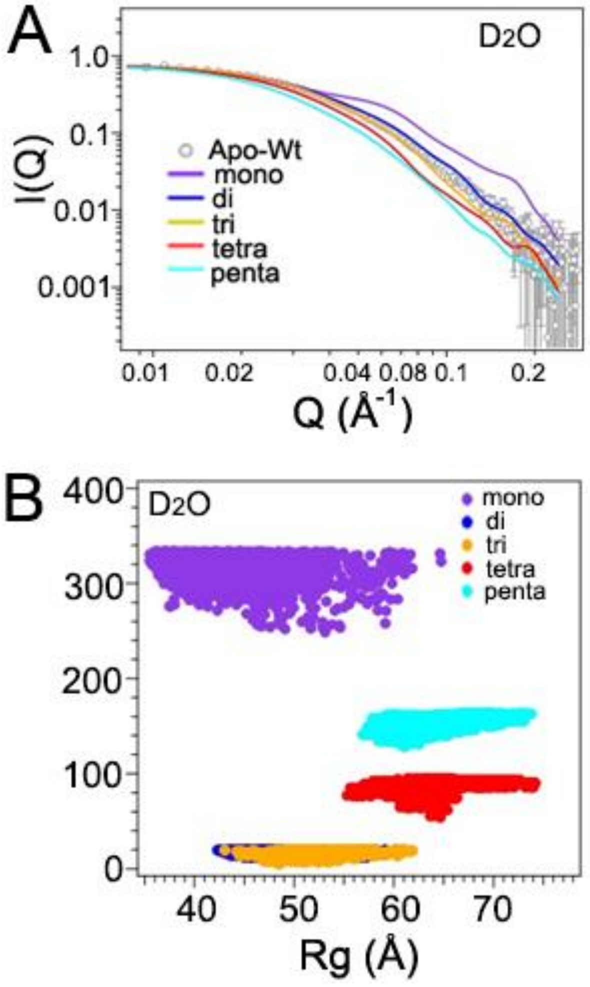

Supplementary Figure S1

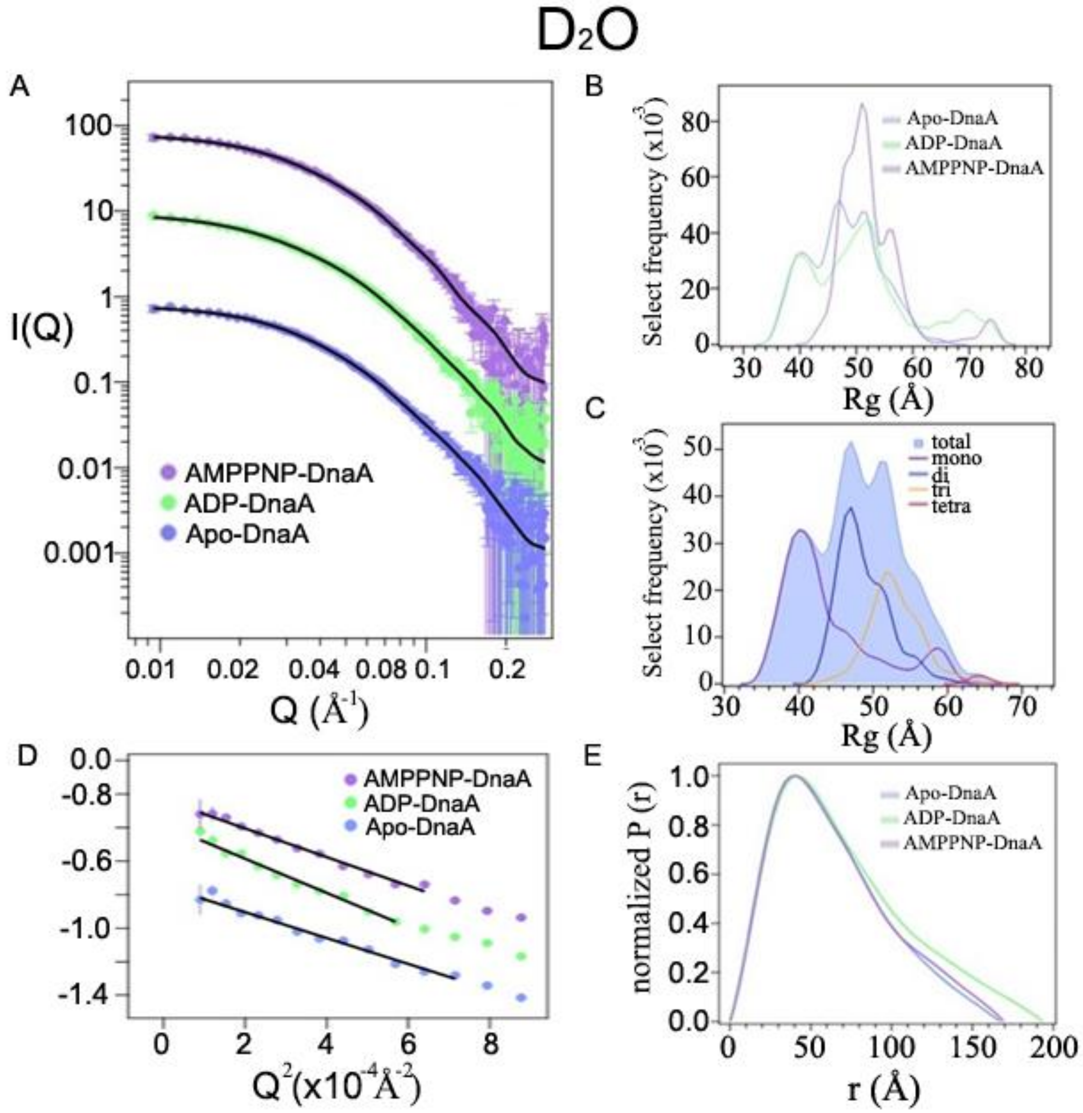

Supplementary Figure S2

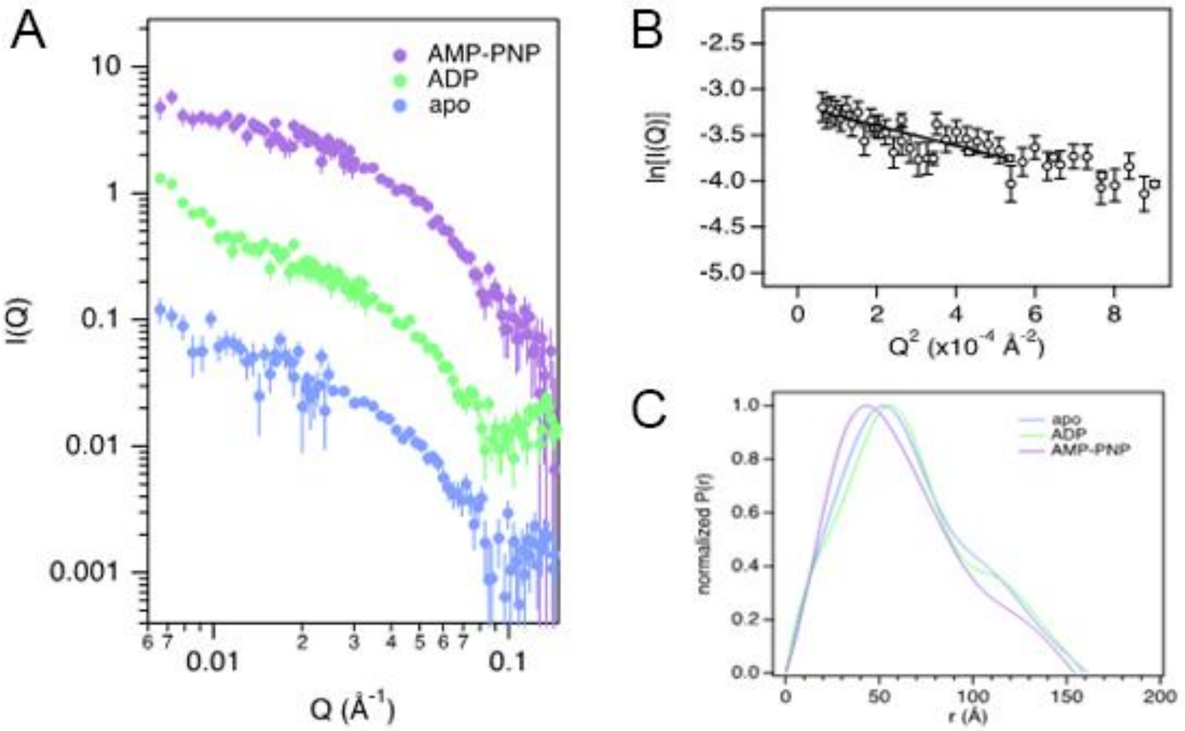

Supplementary Figure S3

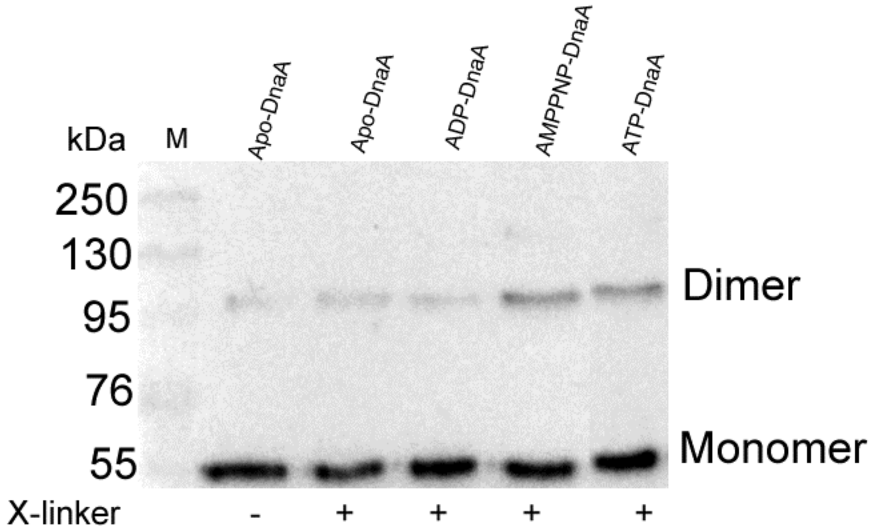

Supplementary Figure S4

```
CLUSTAL 2.1 multiple sequence alignment

E.coli          MSLSLWQQCLARLQDELPATEFSMWIRPLQAELSDNTLALYAPNRFVLDWVRDKYLNNIN
S.enterica      MSLSLWQQCLARLQDELPATEFSMWIRPLQAELSDNTLALYAPNRFVLDWVRDKYLNNIN
K.pneumonia     MSLSLWQQCLARLQDELPATEFSMWIRPLQAELSDNTLALYAPNRFVLDWVRDKYLNNIN
Y.pestis        MSLSLWQQCLARLQDELPATEFSMWIRPLQAELSDNTLALYAPNRFVLDWVRDKYLNNIN
                ************************************************************

E.coli          GLLTSFCGADAPQLRFEVGTKPVTQTPQAAVTSNVAAPAQVAQTQPQRAAP-STRSGWDN
S.enterica      GLLNTFCGADAPQLRFEVGTKPVTQTLKTPVH-NVVAPAQTTTTQPQRVAP-AARSGWDN
K.pneumonia     GLLNDFCGADAPQLRFEVGAKPASSLQKGAVS-PAAAAIPAAQVQTARVAPTIVRPGWDN
Y.pestis        GLLNDFCGTEVPLLRFEVGSKPAARAHNNPVT----ASVSAPVAPVTRSAP--MRPSWDN
                ***. ***::.* ******:**.:   : .*     *.  .. .   * **   *..***
                                                                     WalkerA
E.coli          VPAPAEPTYRSNVNVKHTFDNFVEGKSNQLARAAARQVADNPGGAYNPLFLYGGTGLGKT
S.enterica      VPAPAEPTYRSNVNVKHTFDNFVEGKSNQLARAAARQVADNPGGAYNPLFLYGGTGLGKT
K.pneumonia     VPAPAEPTYRSNVNVKHTFDNFVEGKSNQLARAAARQVADNPGGAYNPLFLYGGTGLGKT
Y.pestis        SPAQPELSYRSNVNPKHTFDNFVEGKSNQLARAAARQVADNPGGAYNPLFLYGGTGLGKT
                 ** .* :****** *********************************************
                                                                 WalkerB
E.coli          HLLHAVGNGIMARKPNAKVVYMHSERFVQDMVKALQNNAIEEFKRYYRSVDALLIDDIQF
S.enterica      HLLHAVGNGIMARKPNAKVVYMHSERFVQDMVKALQNNAIEEFKRYYRSVDALLIDDIQF
K.pneumonia     HLLHAVGNGIVARKPNAKVVYMHSERFVQDMVKALQNNAIEEFKRYYRSVDALLIDDIQF
Y.pestis        HLLHAVGNGIMARKANAKVVYMHSERFVQDMVKALQNNAIEEFKRYYRSVDALLIDDIQF
                **********:***.*********************************************
                                SensorI
E.coli          FANKERSQEEFFHTFNALLEGNQQIILTSDRYPKEINGVEDRLKSRFGWGLTVAIEPPEL
S.enterica      FANKERSQEEFFHTFNALLEGNQQIILTSDRYPKEINGVEDRLKSRFGWGLTVAIEPPEL
K.pneumonia     FANKERSQEEFFHTFNALLEGNQQIILTSDRYPKEINGVEDRLKSRFGWGLTVAIEPPEL
Y.pestis        FANKERSQEEFFHTFNALLEGNQQIILTSDRYPKEINGVEDRLKSRFGWGLTVAIEPPEL
                ************************************************************
                                           SensorII
E.coli          ETRVAILMKKADENDIRLPGEVAFFIAKRLRSNVRELEGALNRVIANANFTGRAITIDFV
S.enterica      ETRVAILMKKADENDIRLPGEVAFFIAKRLRSNVRELEGALNRVIANANFTGRAITIDFV
K.pneumonia     ETRVAILMKKADENDIRLPGEVAFFIAKRLRSNVRELEGALNRVIANANFTGRAITIDFV
Y.pestis        ETRVAILMKKADENDIRLPGEVAFFIAKRLRSNVRELEGALNRVIANANFTGRAITIDFV
                ************************************************************

E.coli          REALRDLLALQEKLVTIDNIQKTVAEYYKIKVADLLSKRRSRSVARPRQMAMALAKELTN
S.enterica      REALRDLLALQEKLVTIDNIQKTVAEYYKIKIADLLSKRRSRSVARPRQMAMALAKELTN
K.pneumonia     REALRDLLALQEKLVTIDNIQKTVAEYYKIKVADLLSKRRSRSVARPRQMAMALAKELTN
Y.pestis        REALRDLLALQEKLVTIDNIQKTVAEYYKIKVADLLSKRRSRSVARPRQMAMALAKELTN
                *******************************:****************************

E.coli          HSLPEIGDAFGGRDHTTVLHACRKIEQLREESHDIKEDFSNLIRTLSS
S.enterica      HSLPEIGDAFGGRDHTTVLHACRKIEQLREESHDIKEDFSNLIRTLSS
K.pneumonia     HSLPEIGDAFGGRDHTTVLHACRKIEQLREESHDIKEDFSNLIRTLSS
Y.pestis        HSLPEIGDAFGGRDHTTVLHACRKIEQLREESHDIKEDFSNLIRTLSS
                ************************************************
```

Supplementary Figure S5

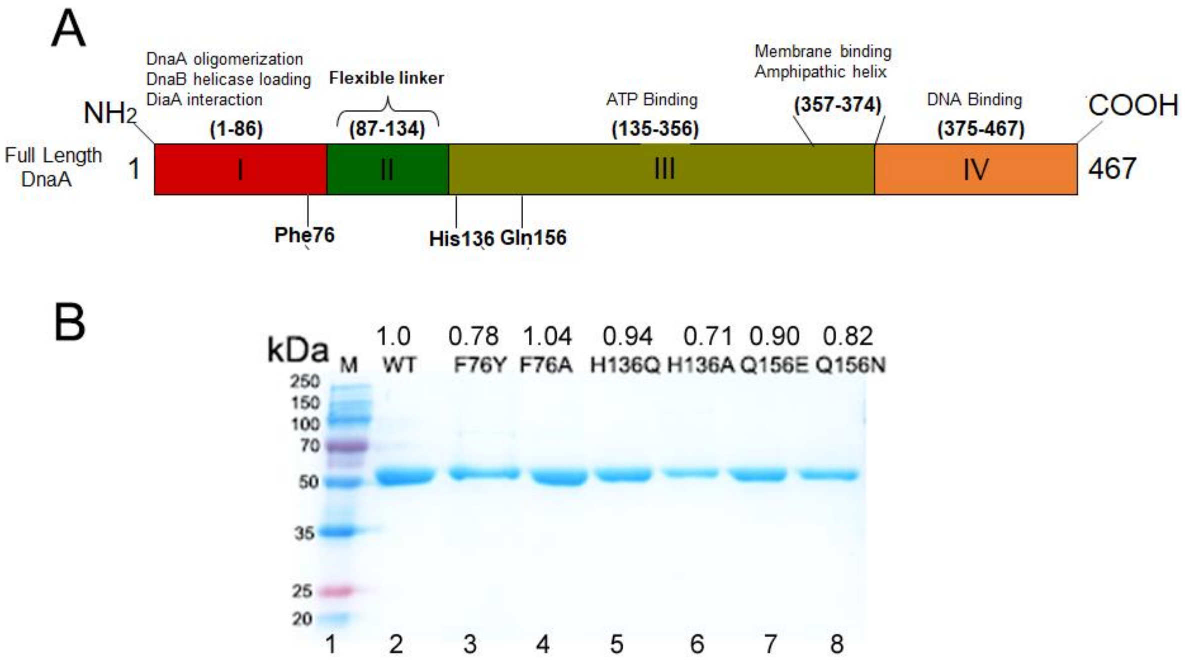

Supplementary Figure S6

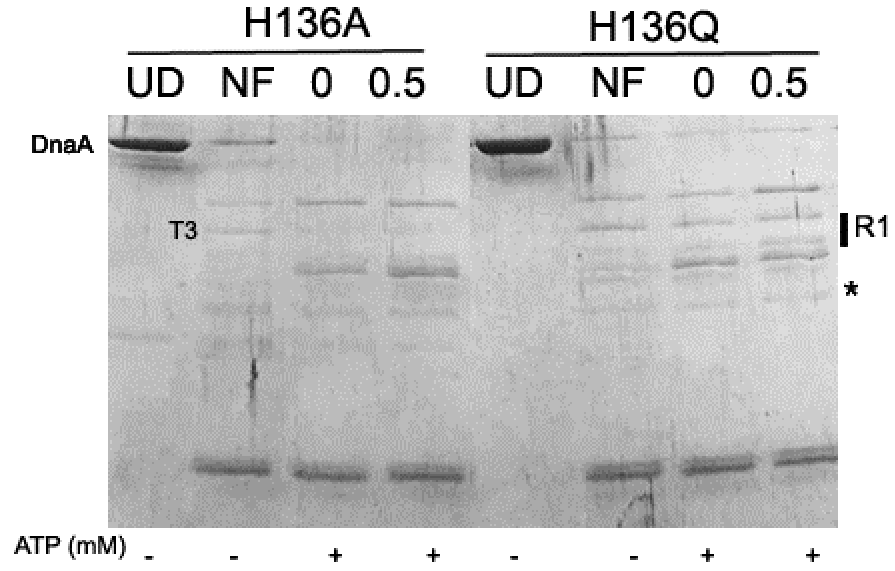

Supplementary Figure S7

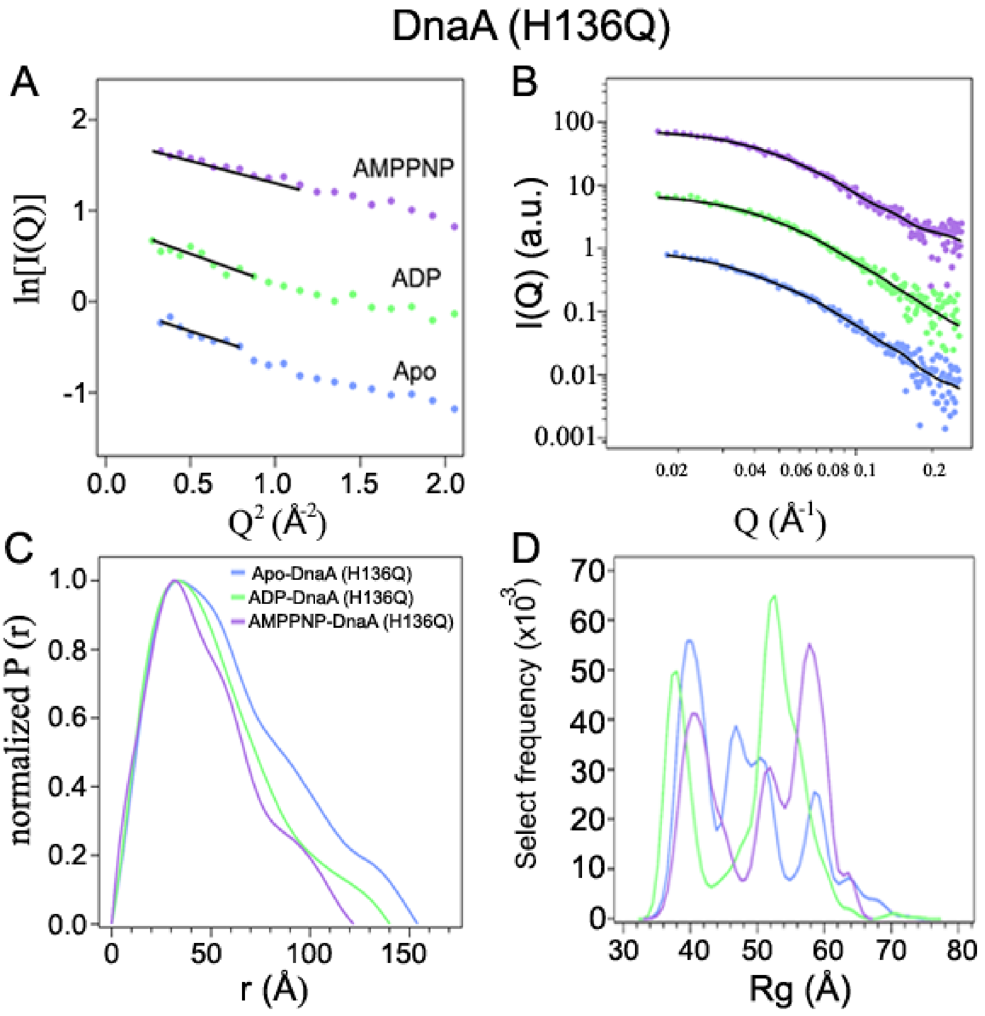

Supplementary Figure S8

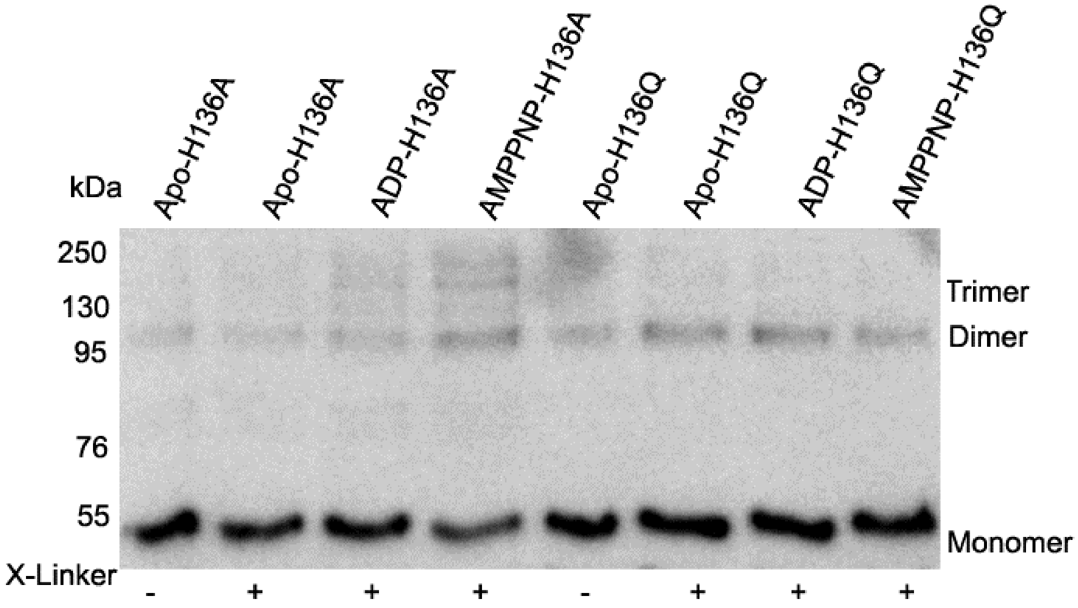

Supplementary Figure S9

Table S1: PCR primers used in the study

Primer name	Primer Sequence (5’ to 3’)	Point mutation
**External primer for generating point mutations in *Escherichia coli dnaA***		
CS4 (sense)	5’ GACAAAGGATCCTTACGATGACAATGTTCTGATT 3’	
CS30 (antisense)	5’ CCAATTAAGCATATGTCACTTTCGCTATGGCA 3’	
**Internal primer for generating point mutations in *Escherichia coli dnaA***		
CS101 (sense)	5’ GCTGCGTTATGAAGTCGGCACC 3’	F76Tyr
CS102 (antisense)	5’ GCCGACTTCATAACGCAGCTGTGG 3’	
CS103 (sense)	5’ GCTGCGTGCTGAAGTCGGC 3’	F76A
CS104 (antisense)	5’ GCCGACTTCAGCACGCAGC 3’	
CS105 (sense)	5’ TTCTAACGTAAACGTCAAAGCCAC 3’	H136A
CS106 (antisense)	5’ GTGGCTTTGACGTTTACGTTAGAA3’	
CS107 (sense)	5’ TTCTAACGTAAACGTCAAACAGAC 3’	H136Q
CS108 (antisense)	5’ GTCTGTTTGACGTTTACGTTAGAA 3’	
CS129 (sense)	5’ CAAAAGGACGTTTGATAACTTCGTTGAA 3’	H136R
CS130 (antisense)	5’ TTCAACGAAGTTATCAAACGTCCTTTTG 3’	
CS109 (sense)	5’ GCGAGGTGGCGGATAACC 3’	Q156E
CS110 (antisense)	5’ CCACCTCGCGAGCCG 3’	
CS111 (sense)	5’ GCGCCGTGGCGGATAACC 3’	Q156A
CS112 (antisense)	5’ GCCACGGCGCGAGCC 3’	
CS133 (sense)	5’ GCTCGCAACGTGGCAGATAAC 3’	Q156N
CS134 (antisense)	5’ GTTATCTGCCACGTTGCGAGC 3’	

*CS4 and CS30 were used as external primers for the amplification of *dnaA* along with mutation. CCAATTAAGCAT in CS30 and GACAAAGGATCC in CR4 do not correspond to genome sequence but has been introduced to incorporate *Nde*I and *BamH I* sites at 5’ and 3’ ends respectively of the PCR amplified product.

Table S2: Plasmids used in the study

Plasmids	Relevant characteristics	Source
pZL411	Amp^r, P*lac* prom, *dnaA*(WT)	Laboratory stock (38)
pRS1	Amp^r, P*lac* prom, *dnaA*(F76Y)	constructed for this study
pRS2	Amp^r, P*lac* prom, *dnaA*(F76A)	constructed for this study
pRS3	Amp^r, P*lac* prom, *dnaA*(H136Q)	constructed for this study
pRS4	Amp^r, P*lac* prom, *dnaA*(H136A)	constructed for this study
pRS5	Amp^r, P*lac* prom, *dnaA*(Q156E)	constructed for this study
pRS6	Amp^r, P*lac* prom, *dnaA*(Q156N)	constructed for this study
pBAD24c	Amp^r, P_{BAD} prom	Addgene
pZL606	Amp^r, P_{BAD} prom, *dnaA*(WT)	Laboratory stock (39)
pRS9	Ampr^r, P_{BAD} prom, *dnaA*(F76Y)	constructed for this study
pRS10	Ampr^r, P_{BAD} prom, *dnaA*(F76A)	constructed for this study
pRS11	Amp^r, P_{BAD} prom, *dnaA*(H136Q)	constructed for this study
pRS12	Amp^r, P_{BAD} prom, *dnaA*(H136A)	constructed for this study
pRS13	Amp^r, P_{BAD} prom, *dnaA*(Q156E)	constructed for this study
pRS14	Amp^r, P_{BAD} prom, *dnaA*(Q156N)	constructed for this study
pAL70	Cm^r *E. coli oriC*	provided by Dr. Julia Grimwade

Table S3: Bacterial strains used in this study

E. Coli Strain	Relevant genotype	Source
BL–21(DE3) pLysS	F– *omp*T, *hsd*SB(rB–, mB–), *galdcm131*(DE3) pLysS (Cam^R)	Invitrogen
DH5α	*rec*A1, *end*A1, *hsd*R17, (rK–,mK+), *pho*A, *sup*E44, *rel*A1)	Invitrogen
EH3827	CM1565 *zia::pKN500* Δ*dnaA mad-1 tnaA::Tn10*	(45)

Table S4. SAXS results for DnaA states

	Guinier	$P(r)$		EOM	
state	R_g (Å)	R_g (Å)	D_{max} (Å)	$\langle R_g \rangle$ (Å)	$\langle D_{max} \rangle$ (Å)
Apo	36 ± 6	42 ± 2	150	50.4 ± 0.2	172.7 ± 0.3
ADP 1μM	35 ± 4	37 ± 2	128	51.2 ± 0.1	178.3 ± 0.5
ADP 0.5 mM	29 ± 4	34 ± 2	111	53.6 ± 0.1	184.7 ± 0.6
AMP-PNP 1μM	30 ± 4	34 ± 1	117	54.5 ± 0.1	187.8 ± 0.1
AMP-PNP 0.5 mM	37 ± 4	36 ± 1	120	56.0 ± 0.2	196.9 ± 0.9
Apo (D_2O)	48 ± 1	50 ± 0.3	167	48.3	169
ADP 0.5 mM (D_2O)	55 ± 1	57 ± 0.5	194	51	183
AMP-PNP 0.5 mM (D_2O)	51 ± 1	51.2 ± 0.4	169	53	198

Table S5. SANS results for DnaA states in 93% D_2O buffer

state	$I(0)$ (cm^{-1})	from P(r) Rg (Å)	Dmax (Å)
Apo	0.050 ± 0.002	52.0 ± 1.0	161.2
ADP	0.039 ± 0.001	52.2 ± 0.9	158.5
AMP-PNP	0.034 ± 0.001	48.5 ± 0.6	154.2

Table S6. SAXS results for H136Q DnaA states

	Guinier	*P*(*r*)		EOM	
state	R_g (Å)	R_g (Å)	D_{max} (Å)	$\langle R_g \rangle$ (Å)	$\langle D_{max} \rangle$ (Å)
Apo	43 ± 9	47 ± 2	154	48.1 ± 0.1	161.6 ± 0.3
ADP 0.5 mM	45 ± 7	41 ± 1	140	49.0 ± 0.1	163.4 ± 0.5
AMP-PNP 0.5 mM	38 ± 4	37 ± 1	122	51.0 ± 0.1	174.3 ± 0.3
